# Which Reasons Do Doctors, Nurses, and Patients Have for Hospital Discharge? A Mixed-Methods Study

**DOI:** 10.1371/journal.pone.0091333

**Published:** 2014-03-13

**Authors:** Dirk T. Ubbink, Evelien Tump, Josje A. Koenders, Sieta Kleiterp, J. Carel Goslings, Fleur E. Brölmann

**Affiliations:** 1 Department of Quality Assurance and Process Innovation, Academic Medical Center, Amsterdam, The Netherlands; 2 Department of Surgery, Academic Medical Center, Amsterdam, The Netherlands; 3 Department of Paediatrics, Academic Medical Center, Amsterdam, The Netherlands; 4 Department of Obstetrics and Gynaecology, Academic Medical Center, Amsterdam, The Netherlands; 5 Department of Trauma Surgery, Academic Medical Center, Amsterdam, The Netherlands; 6 Department of Plastic, Reconstructive and Hand Surgery, St Lucas Andreas Hospital, Amsterdam, The Netherlands; The University of York, United Kingdom

## Abstract

**Background:**

The decision to discharge a patient from a hospital is a complex process governed by many medical and non-medical factors, while the actual reasons for discharge frequently remain ill-defined.

**Aim:**

To define relevant discharge criteria as perceived by doctors, nurses and patients for the development of a standard hospital discharge policy, we collected actual reasons and most pivotal medical and organisational criteria for discharge among all stakeholders.

**Setting:**

A tertiary referral university teaching hospital.

**Methods:**

We conducted a mixed methods analysis, using patient questionnaires, interviews and a focus group with caregivers, and observations during the daily rounds of doctors, nurses and patients during their hospital stay. Fourteen wards of the Surgery, Paediatrics and Neurology departments contributed.

**Results:**

We observed 426 patients during their hospital stay. Forty doctors and nurses were interviewed, and 7 senior nurses attended a focus group. The most commonly used discharge criteria were clinical factors, organisational discharge issues and patient-related factors. A total of 269 patients returned their questionnaires. About one third of the adult patients and nearly half of the children (or their parents) felt their personal situation and assistance needed at home was insufficiently taken into account before discharge. Patients were least satisfied with the information given about what they were allowed to do or should avoid after discharge and their involvement in the planning of their discharge. Thus, besides obvious medical reasons for discharge, several non-medical reasons were signalled by all stakeholders as important issues to be improved.

**Conclusions:**

A set of discharge criteria could be defined that is useful for a more uniform hospital discharge policy that may help reduce unnecessary length of stay and improve patient satisfaction.

## Introduction

Hospital discharge is indicated when a patient is ready for another, usually lower, level of care. This may be the home situation with or without district nursing care, but also a nursing home or rehabilitation centre. To most clinicians it may seem clear that patients can be discharged when their clinical condition is ready, but this appreciation seems neither transparent nor uniform. Assessment of the required level of care or, in other words, the actual reasons for discharge, frequently remain unidentified, poorly documented, or ill-defined [1], [2]. Conversely, it may be equally unclear why a patient cannot be discharged yet.

The decision to discharge a patient from a hospital is a complex process governed by many factors, which comprise not only medical but also organisational reasons, and not all of which are easily controlled. It has been estimated that approximately 30% of hospitalised patients experience a delay of their discharge, while about 30% of these delays are due to non-medical factors [3], [4].

Given these circumstances, early discharge planning has been advocated to improve patient outcomes, quality of care, and hospitals’ logistic and financial concerns [5], [6]. Consequently, considerable attention has been directed towards designing a better discharge policy in many countries worldwide [1], [7], [8]. In the Netherlands, the national Health Care Inspectorate requires early discharge planning and clear discharge criteria. Unfortunately, random inspection showed that is neglected in 83% of the observed hospitals [9]. Moreover, satisfaction among recently discharged patients regarding the timing of discharge tends to score low [10].

Hence, not only the discharge planning process, but rather the formulation of discharge criteria, preferably based on the perception of both clinicians and patients, is needed in order to meet the requirements of standardised discharge planning. Daily, the discussion about when to discharge is repeating itself for thousands of hospitalised patients worldwide. However, literature is conspicuous by its absence regarding established reasons for discharge. Only for specific disorders, like colorectal disease, such criteria have been studied [11], which has resulted in an expert-based consensus [12].

Altogether, a better understanding of the reasons healthcare providers and patients perceive for hospital discharge and the patients’ satisfaction with this process seems a first crucial step to develop a uniform, efficient, and timely hospital discharge policy and ultimately to provide a better quality of the organisation of care. This study was conceived in 2010 when our university hospital had embarked upon a Joint Commission International (JCI) quality accreditation process. One of the JCI-criteria, i.e. regarding the access and continuity of care, requires a uniform, hospital-wide discharge policy. Despite earlier initiatives to formulate explicit discharge criteria, for example within our department of Surgery, and an early discharge policy developed at our department of Internal Medicine, no such document existed. To generate a valuable resource of discharge criteria as a cornerstone of this document, we collected actual medical and non-medical reasons and most pivotal criteria for discharge as perceived by physicians, nurses, and patients from different clinical wards. We also appreciated patients’ preferences for, and satisfaction with, the discharge process.

## Methods

A mixed qualitative and quantitative method was used to appreciate actual and desired discharge criteria and to gauge patient satisfaction with this procedure. The “Consolidated criteria for reporting qualitative research” (COREQ) were used as a reporting framework [13].

### Ethics Statement

The medical ethics review board of the Academic Medical Center at the University of Amsterdam approved the study, but waived the need for the patient’s informed consent, as they judged the study would not have any impact on the patients’ psychological or medical integrity. Hence, no written informed consent was asked from the participants for their clinical records to be used in this study. However, patient records and information were anonymised and de-identified prior to analysis.

### Setting and Contributors

**Figure 1:**
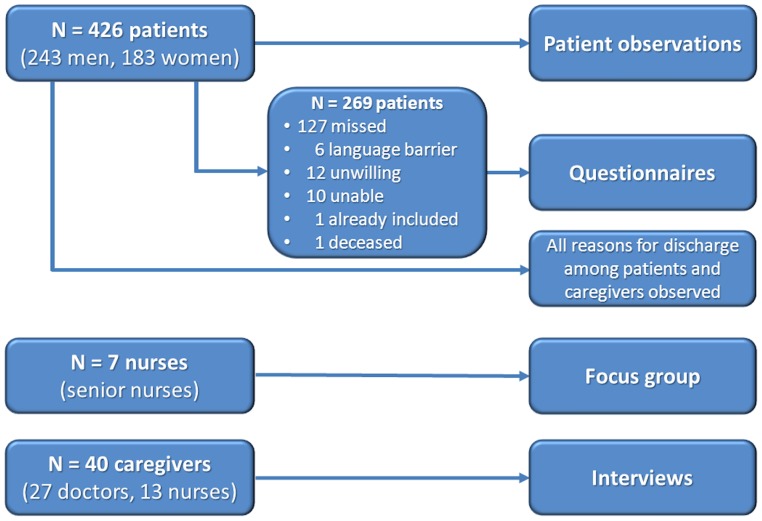
Samples of patients and caregivers investigated.

This study was performed in a tertiary referral university teaching hospital in a period of 7 months (March till September 2011). All 14 wards belonging to the departments of Surgery (n = 8), Paediatrics (n = 5) and Neurology (n = 1) were willing to cooperate. The numbers of patients and caregivers investigated by means of the various qualitative and quantitative methods is shown in Figure 1. At the time of the study no uniform hospital-wide discharge policy existed.

### Qualitative and Quantitative Methods

The qualitative part of the study comprised a focus group, semi-structured in-depth interviews, and non-participatory observations of the daily rounds. We used multiple methods because some reasons for discharge might not surface by mere observations. The personal interviews mainly attempted to gauge organisational reasons for discharge, while the observations during patient rounds focused more on medical and patient-related reasons for discharge. Thus, these different methods allowed triangulation of the data to enhance their completeness and veracity [14]. The focus group and the interviews were carried out during the first weeks of the daily rounds observation period. The topics list for the focus group and interviews was based upon earlier research on discharge planning [5], [6], [15], [16]. During the focus group, interviews, and observations, no other persons than participants and researchers were present who could have influenced these encounters.

For the one-hour focus group session, 7 senior nurses responsible for quality assurance within their wards met to gather and discuss reasons they perceived as relevant for discharge.

For the interviews, we aimed to recruit a purposive sample of at least two doctors and one nurse for each of the 14 wards. We approached professionals with different grades of responsibilities regarding the discharge policy in order to collect all conceivable reasons for discharge. We interviewed twice as many doctors as nurses, because doctors were primarily responsible for setting the moment of discharge. We eventually interviewed a total of 27 doctors (3 professors, 15 consultants/staff specialists, 9 residents) and 13 nurses (10 head nurses and 3 registered ward nurses).

For the qualitative and quantitative observations of the daily rounds, our study sample consisted of 426 patients, admitted to the departments of surgery (N = 230 adults; 54.0%), paediatrics (N = 142 children; 33.3%), and neurology (N = 54 adults; 12.7%). The numbers per (sub)specialty are shown in Table 1. In each department, discharge data from patients were collected consecutively during a period of 2 to 4 weeks, until a sufficient number of patients was reached. “Sufficiency” was determined by data saturation (i.e., until the observations yielded no more new insights) and representativeness (i.e., typical age and gender distribution and types of disorders for that ward or specialty).

**Table 1:** Patient and discharge characteristics.

DEPARTMENT	PatientsN = 426 (100%)	Male gender(%)	Age in years(mean; range)	Hospital stayin days(mean; range)	ASAclassification	Discharge to
**Surgery**	**230 (54%)**	132 (57%)	56.4 (16–94)	7.1 (1–33)	1: 66(29%)	Home 185 (80%)
					2∶108(47%)	Home with home care16 (7%)
					3: 54(23%)	Other hospital 15 (7%)
					4: 2(1%)	Nursing home or rehab11 (5%)
						Other department 3 (1%)
• Gastro-intestinal	61 (14%)					
• Thoracic	38 (9%)					
• Orthopaedic	33 (8%)					
• Urology	28 (7%)					
• Short stay	28 (7%)					
• Trauma	20 (5%)					
• Plastic & reconstr.	14 (3%)					
• Vascular	8 (2%)					
**Paediatrics**	**142 (33%)**	81 (57%)	7.2 (0–19)	5.7 (1–28)	1∶55(39%)	Home 136 (95%)
					2∶59(42%)	Other hospital 5 (3%)
					3∶25(18%)	Nursing home or rehab1 (1%)
					4: 2(1%)	Other department 1 (1%)
Infants	24 (17%)					
• 1–10 years	40 (28%)					
• 11–18 years	41 (29%)					
• Oncology	25 (18%)					
• Surgery	12 (8%)					
**Neurology**	**54 (13%)**	30 (56%)	58.2 (23–85)	11.3 (1–69)	1∶20(37%)	Home 37 (69%)
					2∶15(28%)	Nursing home or rehab11 (20%)
					3∶17(31%)	Other department6 (11%)
					4: 0(0%)	
					Missing: 2(4%)	

Quantitative data were obtained by counting the reasons for discharge mentioned during the daily rounds. Semi-quantitative data were collected by means of a patient questionnaire to appreciate their satisfaction and experience with the discharge process when they were about to leave the hospital (Appendix S1). We extracted relevant questions from validated, nation-wide used questionnaires appreciating general patient satisfaction during hospitalisation [10], [17]. Some of these items were altered to address the discharge rather than the hospitalisation. Each question was to be answered on a three- or five-point Likert scale (Appendix S1).

We first piloted this questionnaire for a period of 2 weeks. Feedback from patients and a local, independent expert in questionnaire research, were used to fine-tune the questionnaire. We received feedback on the size of the questionnaire, and the readability and completeness of the discharge information.

### Procedures

#### Focus group

The focus group was led by one researcher (JK) and field notes were taken by another (DU). First, the aim of the focus group was communicated: ‘exploration of discharge criteria’. Next, in several rounds the attendees were invited to answer the questions: “What reasons should influence discharge” and “Which discharge criteria do you use at the moment?” After each round they were given the opportunity to respond to the reasons brought up by others. This was continued until no more new reasons for discharge surfaced. All motivations and remarks on patient discharge were recorded anonymously. Finally, the attendants were invited to discuss problems occurring at the time of discharge. We specifically asked whether bed occupation or waiting for the outcome of multidisciplinary (oncology) meetings were items influencing the discharge policy. Afterwards, focus group members were free to comment and discuss the gathered list of discharge criteria.

#### Interviews

Semi-structured in-depth interviews took place with doctors and nurses in private rooms to encourage participants to openly convey their viewpoints. The researchers (JK, ET, SK, DU and FB) approached all interviewees personally. The interviews took 20 minutes on average and were digitally recorded. Appendix S2 presents the semi-structured questions used. None of the caregivers were interviewed more than once. Field notes and interviews were transcribed by the researchers (JK, ET, SK and FB) within a week after the interview. Transcripts were not returned to the interviewees for feedback because doctors and nurses were short in time and therefore denied the privilege.

#### Observations

During the seven-month study period, five non-participating researchers (JK, ET, SK, DU, and FB), i.e., one researcher per ward, openly observed the daily rounds on the wards to collect all reasons regarding discharge as mentioned by doctors, nurses, or patients. Throughout the observation period the growing list of occurring reasons was discussed, and adjusted if necessary, at meetings held every two weeks by the five researchers (JK, ET, SK, DU and FB).

Also during these rounds, for each reason mentioned for a particular patient the researchers scored “red” if the reason was mentioned but its current status did not allow for discharge (e.g., patient has an i.v. drip that cannot yet be removed); “green” when the status made discharge possible (e.g., the i.v. drip had been removed); and “white” when the reason was not applicable for a particular patient during the whole hospitalisation period (e.g., the patient did not have an i.v. drip at all). During hospitalisation, applicable reasons changed from “red” to “green”. Thus, the patient could theoretically be discharged when all recorded reasons scored “green”.

The reasons and their scores were recorded as field notes and categorised afterwards in a code tree. These consisted of vital decision-making remarks, responsibilities and actions towards the discharge, questions asked by either caregiver or patient, and other events influencing the decision-making regarding discharge. These units were based on previous literature data and clinical expertise [5], [6], [15], [16].

#### Questionnaires

All observed patients received the final questionnaires (appendix S1) to be completed at the end of their hospital stay. In case of young children, the child’s parents received the questionnaire.

#### Patient data

Patient characteristics were retrieved from the patients’ medical or nursing files. Exact admission and discharge dates were derived from the hospital databases. For all patients, regardless of whether they would undergo surgery, we registered the patients’ American Society of Anesthesiologists classification (ASA; 4 categories), a system for assessing the fitness of patients before surgery [18]. If patients had surgery, we also recorded the technical complexity of the surgical procedure performed, defined according to the surgery complexity scale used by Dutch Surgical Association, ranging from 1, “simple” to 7, “complex” procedures [19].

### Data Analysis

#### Qualitative data analysis

The interviews were transcribed verbatim. While coding the qualitative data, a number of themes was generated, capturing reasons influencing discharge. A code tree was developed using Moons’ definition of discharge management [20]. This code tree contained three themes; 1) “Patient-related discharge reasons”, 2) “Medical devices, laboratory tests, and medical care” and 3) “Organisation-related discharge reasons”. The interviews and field notes were examined for reasons or barriers concerning hospital discharge. Eventually, one theme was added to the initial code tree, based on iterative discussions among the three researchers (JK, ET and FB) : 4) “Physical checks”. For each of these themes a representative view was developed and illustrated by quotes from various contributing healthcare professionals.

To double-check our code tree, three researchers (JK, ET and FB) independently coded the verbatim transcripts of the interviews using MAXQDA software (version 10, VERBI GmbH, Berlin, Germany) [21]. Based on these data, we developed a list of generic and department-specific reasons for discharge, within each of the four themes (Table 2).

**Table 2:** Reasons for discharge, based on interviews and focus group*, and observations during daily rounds, categorised into four themes.

**1.Patient-related reasons**	
General medical and mental condition*	Independent activities of daily living (ADL)*
Food & fluid intake*	Wound shows sufficient healing tendency*
Motor & sensory function	Oedema of surgical site
Bowel/stoma function	Wound self-care*
Artificial (nasogastric) feeding*	Stoma self-care
Artificial (parenteral) feeding*	Anticoagulation administration instructed*
No nausea/vomiting	Pain management*
Fluid balance	Mobility*
Weight	Spontaneous miction*
Drug adverse effect	Parents instructed
Patient (or parents) agree with discharge*	Patient’s questions have (not yet) been answered
Do Not Resuscitate (DNR), Glasgow Coma Scale (GCS),or Delirium Observation Screening (DOS)	
**2) Medical devices, laboratory tests, and medical care**	
Monitor surveillance	Intravenous drip
Nasogastric tube removed	Urine catheter*
Plaster cast	Palliative care arranged
Negative pressure wound therapy (NPWT)*	Medical test (e.g., pre-discharge imaging)
Lab: Urine pH	Lab: electrolytes*
Lab: infection signs (e.g., WBC, CRP)*	Lab: other (e.g., Hb, INR, creatinine, glucose)*
Interdisciplinary discharge plan	Multidisciplinary meeting results*
Medical interventions (e.g., removal of stitches ordrain replacement)*	Other paramedic disciplines consulted(e.g., dietician, physical therapist, social worker)*
Other treating medical disciplines consulted (e.g.,geriatric or internal specialists)*	
**3)** **Organisation-related reasons**	
Outpatient clinic appointment*	Discharge letter and papers*
Medication list and prescriptions*	Situation at home or at follow-up institution*
Arrangements for home care*	Arrangements for transport facilities*
Bed is needed for new admission*	Planned treatment is cancelled*
Discharge during weekends	Insurance issues*
**4) Physical checks**	
Heart rate	Blood pressure
Body temperature	Respiration frequency
Wound drain/leakage*	Urine blood clots
Need for additional oxygen	

#### Quantitative data analysis

The number of times a reason for discharge was applicable in our set of observed patients at the end of their hospital stay (i.e. “green”, or still “red” in a few cases) was counted to arrive at a list of mostly used discharge reasons. Only the valid responses of the questionnaires were used, i.e., after discarding the “don’t know” answers. Satisfaction results were calculated as the percentage of patients who scored “satisfied” or “very satisfied”. For the data analysis, standard descriptive statistics were used. Data analysis was performed using IBM SPSS Statistics (v. 20, IBM, Armonk, NY, USA).

## Results

### Patient Characteristics

We included patients from 8 surgery, 5 paediatric and 1 neurology wards. Details about the included patients (N = 426) and their discharge are presented in Table 1. Two-thirds of the patients were adults. One quarter of the surgical patients did not undergo surgery. The technical complexity levels of the surgical procedures for adult patients performed were: 1 (in 7% of the patients), 2 (9%), 3 (6%), 4 (27%), 5 (20%), 6 (17%) and 7 (15%), in keeping with the tertiary referral level of the hospital.

### Qualitative Analysis

In six rounds the participants of the focus group shared their reasons for discharge. These are detailed in Table 2. The four items that emerged could be categorised into medical and non-medical reasons for discharge. The medical reasons comprised the themes “Patient-related items”, “Medical devices, laboratory tests, and medical care”, and “Physical checks”. Non-medical reasons were captured in the theme “Organisation-related reasons”.

#### Theme “Patient-related items”

“Patient-related items” addressed the intactness, normalisation, or at least return to an acceptable level, of the patient’s body functions before discharge would be feasible. One of the interviewees, a paediatric nurse, formulated the importance of verifying the condition of the patient before discharge:

“*[…] the child cannot leave unless we have seen with our own eyes they are doing well.*”

Not only the interpretation of the patient’s condition, but also of the patient’s expectations needs attention. This was illustrated by a surgeon, saying:

“*So I thqink people and their relatives want to stay longer than we feel necessary*.

*[…] They expect more from the hospital than we offer them.*” (surgeon)

Furthermore, the child’s parents also need proper instructions before discharge:

“*Instruction to parents plays an important role. You must be sure a parent has understood you and can repeat back when to contact the hospital. If you’re not, the child cannot leave.*” (paediatric nurse)

#### Theme “Medical devices, laboratory tests, and medical care”

This theme involved the completion of lab testing or follow-up imaging to check the patient’s condition or the removal of catheters and drains before discharge. Some of these may remain in situ at discharge only if indispensable. Performing some final tests may take additional time, but may be performed quicker as long as the patient is hospitalised, which needs some explanation to the patient:

“*People get annoyed when they have to wait for their MRI. This goes faster when they’re hospitalised than when they are outpatient. If you explain this, they are more understanding.*” (senior nurse)

Additional diagnostic testing may also occur when complications occur during hospitalisation:

“*If a patient acquires a delirium, they cannot go home or to a rehab centre. The geriatrician has to treat them first. Then they stay long, because they have to get over their delirium. And if that’s difficult, you have to consider a neurological cause. Then you are also doing diagnostic tests.*” (neurologist)

#### Theme “Physical checks”

The last theme within the medical reasons for discharge also covered the (routine) assessment of the patient’s condition, particularly when the expert distrusts his ‘gut feeling’:

“*Physical checks, yes, these are really the objective clinical parameters. You sometimes cannot rely on the clinical condition only, […] particularly in dialysis patients. You can miss things because they don’t get a fever and you don’t see anything from the outside”. (surgical resident)*

However, checking everything before discharge was not considered obligatory in all cases:

“*Open wounds can be a problem. Patients find wound care at home annoying. We have been quite defensive in the past; everything has to be safe before a patient could leave. Now we explain the patient: the wound may re-open andthen you just come back*” (professor of surgery)

#### Theme “Organisation-related discharge reasons”

Non-medical or organisational issues were also considered important criteria for discharge, for example when things have to be arranged during weekends:

“*To decide on Friday that someone can go home on Monday with tube feeding and therefore needs home care is just not possible, because there is no one to arrange these things during the weekends.*” (nurse)

Besides clinical reasons for discharge, other factors like bed occupation appear to play a variable role when deciding whether a patient should be discharged:

“*When I’m honest and it is busy and we need to admit many patients we look more scrupulously, are more alert than when it is quiet, because you feel that pressure. I think there’s no getting away from that*…”. (paediatrician)

The reasons for discharge as perceived by the focus group participants could all be verified by the information from the personal interviews and observations. The interviews added “discharge during weekends” as a particular issue, while the observations yielded several other medical reasons for discharge. In addition, the participants of the focus group stated that discharge planning usually started late during hospitalisation and without using discharge checklists, if any, except for the at-discharge part of the SURPASS^©^ checklist [22]. Moreover, the amount of information given to the patient and the attention to the patient’s medication were perceived as showing room for improvement.

Based on the focus group, interviews of caregivers, and observations during visit rounds, a final set of discharge criteria was produced (Table 2), categorised into the four themes. Medical reasons for discharge (based on the patient’s condition, lab tests and physical checks) covered most of the criteria as derived from the daily rounds and focus group. Non-medical and organisational reasons formed another important group of criteria, mainly mentioned in the personal interviews and patient questionnaires. Both groups of criteria were subsequently used to be counted in the quantitative analysis.

### Quantitative Analysis

The most frequently used discharge criteria in the various departments, defined as those used in at least 50% of the patients, are shown in Table 3. Overall, the most commonly used criteria were: *clinical criteria* (patient’s clinical condition, body temperature, blood pressure, oxygen saturation, removed iv-drip), *discharge criteria* (outpatient appointment, discharge letter, medication list) and *patient-related criteria* (pain management, (un)answered questions, and agreement as to discharge). Items from the themes “medical devices, lab tests, and medical care” and “other” emerged less frequently as relevant discharge criteria.

**Table 3:** Most frequently used discharge criteria per patient, as applied in the various departments (in bold department-specific criteria not occurring in the overall list, in italic the non-medical reasons).

	Overall (n = 426)	%	Surgery (n = 230)	%	Paediatrics (n = 142)	%	Neurology (n = 54)	%
**1**	Body temperature	98	Body temperature	100	Body temperature	94	Body temperature	100
**2**	Blood pressure	90	Blood pressure	100	**Heart rate**	88	Blood pressure	100
**3**	Clinically well	84	*Patient agrees*	93	**Oral intake**	78	Discharge letter	100
**4**	*Outpatient appointment*	76	Clinically well	90	Clinically well	76	Oxygen saturation	100
**5**	Pain under control	75	Infusion (iv/port-a-cath)	89	*Outpatient appointment*	73	**Lab: electrolytes**	100
**6**	Oxygen saturation	73	Pain under control	88	***Family agrees***	73	**Drain**	100
**7**	Infusion (iv/port-a-cath)	72	*Outpatient appointment*	81	*Questions answered*	71	*Patient agrees*	98
**8**	*Patient agrees*	70	Oxygen saturation	80	Blood pressure	70	Clinically well	80
**9**	*Discharge letter*	67	*Medication list*	69	Pain under control	68	***Home situation ready***	67
**10**	*Medication list*	59	*Discharge letter*	59	*Discharge letter*	68	***Family agrees***	65
**11**	*Questions answered*	51	**Lab: haematology**	58	Infusion (iv/port-a-cath)	64	*Outpatient appointment*	61
**12**			**ADL capable**	57	*Medication list*	63	***Other disciplines involved***	56
**13**			*Questions answered*	50	**Respiration frequency**	60	**Additional diagnostics**	52
**14**					Oxygen saturation	52	**Mobility**	52
**15**					***Other disciplines involved***	52		

Overall, 5 out of the 11 most frequently used discharge criteria were non-medical reasons; appointment made at the outpatient clinic, discharge letter supplied, medication list provided, patient agrees with discharge, and his or her questions have been answered.

Relatively few additional department-specific criteria were found to be important, i.e., the differences in discharge criteria between the specialties were small. Some criteria were particularly relevant to a certain specialty, e.g., the need for a subsequent care institution (in Neurology, Orthopaedic or Vascular Surgery), the ability to perform activities of daily living (Orthopaedics and Vascular Surgery), or signs of an infection (GI-surgery).

In 12% (53/426) of the patients who were discharged, one or more items were scored as “red”; not yet ready for discharge (Table 4). For only 8 out of these 53 patients the reason belonged to the overall top-12 of most mentioned reasons (i.e., patient not yet clinically well and discharge letter not ready). The deciding reason(s) the patients were discharged anyway could not be traced. Some of the remaining “red flags” at discharge suggested that in some cases there may have been a gap between patients’ and caregivers’ perception regarding readiness for discharge. These “red flags” mostly concerned different interpretations of the readiness of the home situation for discharge, in which case a – sometimes temporary - solution was sought. In 4 out of the 426 patients discharge criteria could not be recorded completely because the patients died during hospitalisation; 3 on the Neurology ward, and 1 on the Thoracic Surgery ward.

**Table 4:** Discharge criteria at surgery, paediatric, and neurology wards (in % of patients in whom these criteria were deemed relevant) ordered in the four themes.

SPECIALTY	SURGERY								PAEDIATRICS					NEUROLOGY
CRITERION	Gastro-intestinal	Thoracic	Ortho-paedics	Urology	Shortstay	Trauma	Plastic &reconst.	Vascular	Infants	1–10years	11–18years	Onco.	Surg.	Neurology
Clinically well	96	50^1^	100	100	93	100	100	100	17	65	98	100	100	80^1^
Patient agrees	98	66	100	100	100	95	86	100	0	0	66	20	0	98
Family agrees	0	3	0	0	0	10	0	13	13	65	90	100	100	65^2^
Parents instructed	0	0	0	0	0	0	0	0	0	0	5	0	17	0
ADL capable	90	11^3^	100	4	39	90	14	100	0	0	17	4	0	41
Oral intake	95	0	0	4	25	15	0	38	79	63	81	96	75	4
Mobility	2	47	100	4	36	75	0	38	0	10	37	4	8	52
Motor & sensory function	2	0	100	0	0	60	7	25	0	13	27	4	17	44
Pain under control	97	45	100	96	86	100	93	100	21	65	76	96	92	35
Questions answered	33	0	91	96	11	100	50	100	0	58	100	100	100	0
Drug adverse effect	0	8	0	0	0	0	0	0	4	0	2	92	0	2
Oedema surgical site	0	3^4^	21	0	4	25	29	0	0	8	0	0	0	0
DNR/GCS/DOS	3	11	9	0	0	35	0	0	4	13	7	0	17	33
Fluid balance	2	13^5^	0	0	0	10	0	0	21	35	24	28	42	4
No nausea/vomitus	64	8	3	4	14	5	7	0	29	5	56	96	50	7
Weight	2	84	0	0	0	0	0	0	96	0	2	0	0	0
Total parenteral nutrition	0	0	0	0	0	0	14	0	13	0	0	4	25	0
Stoma care	21	0	0	4	0	0	0	0	0	3	5	0	17	0
Wound care	20	11	67	21	46	65	21	50	4	5	20	4	33	0
Miction spontaneous	2	5	0	89	0	0	7	0	54	13	39	100	67	4
Stool/stoma spontaneous	93	26	3	7	11	20	21	0	58	20	49	72	33	2
Monitor surveillance	0	71^6^	0	0	0	0	0	0	58	0	0	0	0	0
Intravenous treatment	0	13	0	0	0	10	0	0	25	15	10	96	33	19
Urine catheter	80	32	21	71	11	20	21	38	4	3	5	4	0	9
Nasogastric tube	22	3	0	0	0	5	0	0	42	8	10	44	25	6
Drain	36	24	0	7	14	10	7	0	4	0	5	4	0	100
Plaster cast	2	0	24	0	0	5	14	0	0	10	7	0	0	0
Negative Pressure WT	0	0	6	4	0	5	7	0	0	3	0	0	0	0
Lab: infection	100	13	9	14	43	35	14	50	39	23	22	20	17	19
Lab: electrolytes	98	0	21	57	36	20	0	13	0	25	27	92	33	100
Lab: haematology	98	47	64	57	32	30	14	13	71	33	32^7^	92	25	15
INR regulated	0	11	0	0	7	10	7	63	4	0	0	0	0	4
Diabetes regulated	0	0	0	0	0	0	0	0	0	0	0	0	0	7
Urine pH	0	0	0	0	0	0	0	0	0	0	0	40	0	0
Intervention(s) done	11	13	12	14	11	10	0	0	17	0	12	0	0	7
Additional diagnostics	13	76	88	21	11	83	0	13	46	50	32	40	5c0	52
Multidisciplinary meeting	7	0	0	0	0	0	0	0	4	0	0	8	10^8^	0
Palliative care arranged	0	0	0	0	0	0	0	0	0	0	0	1^9^	0	11
Other disciplines involved	25	18	3	11	21	35	14	13	54	60	83	8	8	56
Paramedics involved	23	3	88	0	7	50	0	13	33	8	42	16	8	26
Interdisciplinary plan	5	0	0	0	4	15	0	13	8	3	15^10^	4	0	35
Outpatient appointment	100	3	100	96	93	95	79	100	4	95	78	92	83	61
Medication list	61	53	100	93	43	100	21	100	0	65	73	96	83	2
Discharge letter	95	0	100	4	4	100	100	100	0	60	88^11^	96	100	100
Transport arranged	7	0	15	0	8	25	0	13	25	8	0	0	0	0
Homecare arranged	30	3	21^12^	7	25	25	14	25	8	5	10	96	17	7
Home situation ready	93^13^	5	24	0	96	5^13^	21	25	8	5^13^	5	0	17^13^	67^13^
Awaiting next institution	0	0	0	0	0	15	0	13	4	3	0	0	0	30
Bed problem	0	0	0	0	0	0	21	0	0	0	1	4	0	0
Cancelled surgery	2	0	0	0	7	0	7	0	0	0	2	0	0	0
Insurance issues	0	0	6	0	0	10	0	0	0	0	2	0	0	0
Weekend discharge	3	0	0	4	0	0	0	0	0	3	0	0	0	0
Heart rate	0	100	0	0	0	0	0	0	100	75	85	96	100	0
Blood pressure	100	100	100	100	100	100	100	100	21	58	85	96	100	100
Body temperature	100	100	100	100	100	100	100	100	100	98	85	96	92	100
Oxygen saturation	100	100	100	0	100	30	100	50	58	70	44	8	100	100
Respiration frequency	0	0	0	0	0	0	0	0	100	75	44	12	83	0
Wound leakage	2	18	91	18	0	65	7	13	0	3	12	0	33	0
Urine blood clots	2	0	0	50	0	0	0	0	0	2	0	0	0	0

**In 53/426 (12%) of the patients one or more items were scored as not ready for discharge:**

1 Not considered clinically well enough (n = 6 in Neurology and n = 1 in Thoracic Surgery).

2 Family did not agree (n = 1).

3 Considered not yet able to perform ADL (n = 1).

4 Swelling of surgical site was still prominent (n = 1).

5 Fluid balance was still uneven (n = 1).

6 Discharged with telemetry (n = 1).

7 Low haemoglobin (n = 1).

8 Patient was discharged although results of multidisciplinary oncology meeting were being awaited (n = 1).

9 Palliative care not arranged during hospitalisation (n = 1).

10 Interdisciplinary plan not ready (n = 1).

11 Discharge letter not ready (n = 1).

12 Homecare not available (n = 5 in Orthopaedics and n = 1 in Vascular Surgery)≃ nursing home arranged instead.

13 Home situation not sufficient (Neurology n = 20≃ Trauma Surgery n = 4≃ GI-surgery n = 3, Paediatric s n = 2).

### Questionnaires

A total of 269 questionnaires were returned, 68% of these were from surgical patients. Many patients had already left the hospital without having completed the questionnaire. Other reasons are given in Figure 1. The smaller group of patients who filled out the questionnaires did not differ from the characteristics of the total group of 426 patients. The results are shown in Table 5. In general, patients were satisfied with their discharge process: the overall mean marks ranged between 7.0 (Vascular Surgery) and 8.6 (Urology). The lower mark seemed to coincide with the patients’ perception of a limited amount of information they received and the little influence they had on the discharge planning, albeit that only 7 vascular patients responded. On the other hand, the Vascular and Trauma Surgery wards scored high as to the information about problems that might occur after discharge, while the parents of infants were relatively less satisfied with the information supply.

**Table 5:** Patient satisfaction about hospitalisation and discharge process (part a), information received (part b), and discharge planning aspects (part c) (highest and lowest scores per item are shown in bold and italic, respectively).

SPECIALTY:	SURGERY									PAEDIATRICS						NEUROLOGY
	(N = 182; 68%)									(N = 62; 23%)						(N = 25; 9%)
**ITEM:**	**Gastro-** **intestinal**	**Thoracic**	**Ortho-** **paedics**	**Urology**	**Short** **stay**	**Trauma**	**Plastic &** **reconst.**	**Vascular**	weightedmean	**Infants**	**1–10** **years**	**11–18** **years**	**Onco.**	**Surg.**	weightedmean	**Neurology**
**Patients: N = 269 (100%)**	45	26	29	23	25	14	13	7		15	20	9	12	6		25
**Part a**																
**Overall satisfaction (mean)**	7.9	7.4	8.3	**8.6**	7.8	7.5	7.9	*7.0*		7.9	8.2	8.7	7.7	8.5		8.0
**(range)**	5–10	2–10	5–10	5–10	5–10	3–10	1–10	1–10		2–10	6–10	8–10	5–10	6–10		4–10
**Satisfied with discharge date**	84%	91%	86%	91%	*80%*	86%	85%	**100%**	86.5%	85%	95%	**100%**	88%	**100%**	92.4%	88%
**Satisfied with information given**	96%	95%	81%	91%	91%	92%	75%	*67%*	86.8%	75%	90%	**100%**	80%	83%	85.2%	89%
**Hospitalisation met expectations**	80%	91%	96%	95%	79%	92%	85%	**100%**	90.0%	*69%*	79%	**100%**	80%	83%	80.2%	83%
**Part b**																
**Informed about problems that** **may occur**	77%	83%	76%	86%	83%	**100%**	58%	**100%**	81.0%	*54%*	61%	44%	75%	83%	61.7%	71%
**Contact person given in case** **of problems**	89%	85%	83%	91%	**100%**	79%	77%	71%	86.9%	73%	*68%*	78%	92%	**100%**	78.4%	68%
**Informed about activities to be** **avoided or to do**	50%	54%	86%	**100%**	67%	83%	75%	50%	69.3%	*46%*	58%	67%	50%	40%	53.1%	50%
**Informed about discharge medication**	83%	75%	96%	63%	85%	93%	92%	**100%**	83.7%	*47%*	68%	67%	82%	83%	66.9%	73%
**Relevant caregivers informed**	53%	81%	68%	53%	50%	67%	70%	**100%**	63.1%	44%	58%	80%	*33%*	80%	55.1%	74%
**Part c**																
**Tentative discharge date discussed**	44%	54%	72%	46%	48%	64%	69%	43%	54.0%	73%	65%	56%	**83%**	75%	70.1%	*32%*
**Personal situation considered**	57%	61%	76%	**88%**	72%	76%	60%	75%	68.9%	56%	61%	75%	*33%*	687%	54.3%	80%
**Influence on length of stay**	36%	17%	42%	**86%**	35%	44%	54%	*0%*	38.5%	42%	50%	56%	44%	67%	49.4%	24%
**Assistance needed at home discussed**	52%	39%	**87%**	70%	57%	82%	*20%*	75%	59.6%	67%	56%	75%	22%	67%	55.9%	39%
**Patient feels ready for discharge**	71%	79%	71%	86%	72%	75%	84%	67%	75.3%	92%	90%	**100%**	83%	83%	89.9%	*63%*
**Willingness to help with discharge**	84%	*71%*	91%	91%	80%	89%	82%	**100%**	82.3%	73%	88%	**100%**	90%	83%	86.0%	70%

The hospital stay met the expectations of the vast majority of adult patients (79–100%), while the parents of infants were somewhat less satisfied (69%). Patient satisfaction about the patients’ influence on the length of stay was quite variable among the wards, ranging from 0% to 86%. About one third of the adult patients and nearly half of the children (or their parents) felt their personal situation and assistance needed at home was insufficiently taken into account by their caregivers before discharge. Furthermore, patients were least satisfied with the information provided about what they were allowed to do or should avoid after discharge and their involvement in the planning of their discharge.

## Discussion

This study presents an inventory and quantitative exploration of the medical and non-medical criteria for discharge, as perceived by patients, doctors and nurses, in various specialties in a large university hospital. The most commonly used criteria are suited to be incorporated in a standard discharge policy and can be useful in early discharge planning. For this purpose, not only medical and organisational criteria, but also patient-relevant issues should be involved to assess the optimum moment of the discharge by mutual agreement between caregivers and patients.

Up to now, reasons for discharge are usually considered implicitly by doctors and nurses, while the voice of the patient in this process is inconsistently taken into account. This may lead to undesired variation in care and may impact patient satisfaction and quality of care. Although the notion of early discharge planning has its merits and receives increasing attention [5], [23], it pays less attention to the complex process of decision-making regarding the optimum moment for a patient’s discharge. This study makes explicit the medical and non-medical issues involved in this process.

We did not observe obvious gaps among the stakeholders as to their perception of reasons for discharge, but doctors tended to focus more on the medical condition, nurses on the home situation and discharge procedures, and patients on the information they received and their personal situation. Most of the discharge items that needed the most attention according to the doctors and nurses matched the patients’ perceptions and expectations. This was particularly true for non-medical reasons like (early) involvement of patients (and family) in the discharge process, adequate information supply to the patient at discharge, and a better preparation for, and readiness of, the home situation. These issues are now specifically addressed in the hospital-wide discharge procedure protocol featuring a checklist including these items.

A delayed hospital discharge as well as readmissions due to a premature discharge may be prevented by assessing the patients’ discharge readiness [24], and by employing a standard (early) discharge policy, even in acutely admitted older adults [23], [25], although its effect on health outcomes is still uncertain [5]. Timely assignment of an estimated discharge date is difficult, but can be facilitated by using the Charlson index, which helps identifying patients who are more likely to experience a delay [26]. Our study results as well as previous studies suggest that system-related issues prolong hospital stay at least as much as the severity of illness does [27]. Various psychological and socioeconomic barriers may prevent discharge when the patient is frail, lives alone, or needs a rehab or nursing home facility, which takes time to arrange. Hence, an early discharge planning program is pivotal and should at least comprise several organisational aspects; arranging the outpatient appointment, home care, and a timely preparation of a medication list, discharge letter, and the transport to home or a subsequent care institute.

Also the quality of the discharge process can be improved, particularly when a hospital desires to comply with the Joint Commission International hospital accreditation standard regarding access to, and continuity of, care [28]. For example, a better information supply regarding what to do or to avoid after discharge is often neglected, as well as shared decision-making with the patient regarding the moment of discharge. In addition, the doctor should inform the patient about the anticipated duration of hospitalisation and what they can expect already in the outpatient setting before admission, which is particularly relevant when fast track programs are pursued [7]. Also, problems that might occur after discharge should be identified during the discharge planning process [29], particularly at this juncture where hospitalisation durations are kept at a minimum and health is not completely restored at discharge, for example when wound care needs to be continued [30].

### Strengths and Limitations of this Study

Apparently, all relevant discharge criteria could be collected by triangulation of the various methods applied. The criterion “patient clinically well” seems a rather ill-defined catch-all term, but was used regardlessly. The relatively low number of remaining ‘red flags’ at discharge suggests that the criteria found were indeed relevant. Based on the common criteria found in this study, some organisational improvements were detected to achieve a more efficient discharge procedure. Until recently, different discharge protocols were in use at various departments within our hospital. Progress was made through the introduction among surgical specialties of the SURPASS^©^ checklist [22], which includes several of the most frequently used items we found here. The criteria and improvements as found in the present study will foster the incorporation of a hospital-wide (early) discharge protocol.

Although we collected all reasons mentioned for discharge until saturation was reached, our sample size may have been too small to detect rare reasons for discharge. The frequency or apparent hierarchy (as shown in Table 3) of the reasons for discharge may not necessarily reflect their importance. However, by means of the mixed methods technique, we also found the most relevant reasons for discharge, which we have included in our inventory (Table 2).

About half of the patients studied were from surgical wards. This study started at an inconvenient moment for some other departments as they were in the process of reorganisations at that time. This might hamper the external validity of our results. On the other hand, the discharge criteria were quite similar among the different specialties and seemed little age- or disease-specific. Apparently, the criteria we found among many surgical patients appear to be generic and applicable to a wide range of clinical specialties.

In our study the number of patients completing the questionnaire was substantially lower than those investigated for discharge criteria. This discrepancy was mostly due to the fact that the investigators were notified late about the patients’ discharge and patients desired to leave the hospital right away.

The hospital setting could have influenced the duration of the interviews, since professionals were approached during work hours. Yet, we believed the practical advantages of higher participation levels of professionals outweighed this disadvantage.

We did not specify in our analyses the case-mix of our hospitalised patients. This may differ from other, non-university hospitals, which may in turn limit the generalisability of our results. However, the reasons for discharge found do not seem to be specific to tertiary referral hospitals only.

## Conclusion

This study generated a set of generic discharge criteria, generated by both caregivers and patients, that may be useful for a more uniform hospital discharge policy, which can be incorporated by policy makers in any clinical department or hospital. The set includes both medical, organisational, and patient-oriented aspects and may help reduce unnecessary length of stay and improve patient satisfaction. The formulation of explicit discharge criteria based on the caregivers’ expertise and the patients’ preferences will likely enhance the quality of hospital care and patient satisfaction. Further research may focus on the impact of this discharge policy on the length of hospital stay and patient and caregiver satisfaction.

## Supporting Information

Appendix S1 **Patient questionnaire, to be completed just before discharge (translated from Dutch).**
(DOC)

Appendix S2 **Questions in the semi-structured interviews with doctors and nurses.**
(DOC)
