# Supplementary material for: Which Reasons Do Doctors, Nurses, and Patients Have for Hospital Discharge? A Mixed-Methods Study
Source: PLoS One. 2014 Mar 13;9(3):e91333. doi: 10.1371/journal.pone.0091333 (PMC3953385; doi:10.1371/journal.pone.0091333)
Supplement: Appendix S1 — Patient questionnaire, to be completed just before discharge (translated from Dutch). (DOC) [file pone.0091333.s001.doc]

**Appendix S1.** Patient questionnaire, to be completed just before discharge (translated from Dutch).

1. **Was a tentative discharge date discussed with you during your hospitalisation?**

❑ No

❑ Yes
❑ Don’t know (anymore)

1. **Have relevant persons or institutions (for example: general practitioner, nursing home, rehabilitation centre) been informed by the hospital about your discharge?**

❑ No

❑ Yes
❑ Don’t know (anymore)

1. **Did you receive information about you (new) medication(s) to be used after discharge?**

❑ No

❑ Yes
❑ Don’t know (anymore)

1. **Did you receive information about possible problems or complaints that might occur after discharge (for example: pain, fever, wound infection)?**

❑ No, not at all

❑ Yes, but very little

❑ Yes, some

❑ Yes, certainly

❑ Don’t know (anymore)

1. **Were you told whom you can contact in case of problems or complaints after discharge?**

❑ No

❑ Yes

- Don’t know (anymore)

1. **Did you receive instructions about which activities you could, or should not, do after discharge?**

❑ No, not at all

❑ Yes, but very few

- Yes, some
- Yes, certainly

❑ Don’t know (anymore)

1. **Do you feel healthy and independent enough to leave the hospital?**

❑ No, not at all

❑ Yes, a little

- Yes
- Yes, certainly

❑ Don’t know

1. **Could you ,influence how long you could stay after your treatment or surgery in the hospital?**

❑ No, not at all

❑ Yes, but slightly

❑ Yes

- Yes, certainly
- Don’t know (anymore)

1. **Did your doctor take into account your personal situation when deciding about your discharge date?**

❑ No, not at all

❑ Yes, somewhat

❑ Yes

- Yes, certainly
- Don’t know (anymore)

1. **Did you discuss with the hospital personnel the assistance you might need after discharge?**

❑ No, not at all

❑ Yes, but briefly

❑ Yes

- Yes, certainly
- Don’t know (anymore)

1. **Were your caregivers willing to help you with problems related to your discharge?**

❑ No, not at all

❑ Yes, somewhat

❑ Yes

- Yes, certainly
- Don’t know (anymore)

1. **Was the result of your hospitalisation in agreement with your expectations?**

❑ No, not at all

❑ Yes, somewhat

❑ Yes

- Yes, completely
- Don’t know

**SATISFACTION QUESTIONNAIRE**

1. **How satisfied are you with your date of discharge?**

❑ ❑ ❑ ❑ ❑

Not applicable Dissatisfied Somewhat Satisfied Very satisfied

1. **How satisfied are you with the information you received about further treatment, medication, advice about lifestyle, activities and work, etc.?**

❑ ❑ ❑ ❑ ❑

Not applicable Dissatisfied Somewhat Satisfied Very satisfied

1. **How satisfied are you in general about your discharge? Indicate this with a mark on the scale below:**

❑ ❑ ❑ ❑ ❑ ❑ ❑ ❑ ❑ ❑

Extremely dissatisfied 1 2 3 4 5 6 7 8 9 10 Extremely satisfied

**OPEN QUESTIONS**

**16. What do you think can be improved about your discharge?**

**17. What do you think went well about your discharge?**
