# Supplementary material for: Which Reasons Do Doctors, Nurses, and Patients Have for Hospital Discharge? A Mixed-Methods Study
Source: PLoS One. 2014 Mar 13;9(3):e91333. doi: 10.1371/journal.pone.0091333 (PMC3953385; doi:10.1371/journal.pone.0091333)
Supplement: Appendix S2 — Questions in the semi-structured interviews with doctors and nurses. (DOC) [file pone.0091333.s002.doc]

**Appendix S2.** Questions in the semi-structured interviews with doctors and nurses.

- Does your ward have a discharge protocol?
- How is this discharge policy functioning?
- What would be reasons for this (good or bad) functioning?
- Do you think patients are satisfied with the current discharge policy?
- What do you think are accelerating or decelerating factors for discharge?
- Are there ways to accelerate patient discharge?
- How is the discharge date set: At admission, during hospitalization, after surgery?
- What have been difficult situations regarding patient discharge?
- In particular, are there any logistic reasons (e.g. “wrong beds” or bed occupancy) for a delayed discharge?
- Are there circumstances in which you would prefer to keep a bed occupied for another day to have a bed available for an acute admission?
- Does a uniform discharge policy exist? Is this being discussed in case of disagreements or ambiguity?
- On which criteria do you decide whether a patient can be discharged?
- Do you have any other things to say that might improve the discharge policy?
- We have developed a list of possible reasons for discharge. Could you give us your opinion about this list?
